# Quebracho

**Published:** 1881-06

**Authors:** 


QUEBRACHO.—The results of recent experience with this drug in Bellevue Hospital have been confirmatory of its value in dyspnœa in all its forms. The fluid extract, in doses of from twenty to sixty drops, every hour or two, as called for by the symptoms, has been found useful in our hands also, without regard to the exciting cause of the dyspnœa.

